# Observed changes in wet days and dry spells over the IGAD region of eastern Africa

**DOI:** 10.1038/s41598-023-44115-5

**Published:** 2023-10-06

**Authors:** Paulino Omoj Omay, Nzioka J. Muthama, Christopher Oludhe, Josiah M. Kinama, Guleid Artan, Zachary Atheru

**Affiliations:** 1 Department of Earth and Climate Sciences, Faculty of Science and Technology, University of Nairobi, Nairobi, Kenya; 2 IGAD Climate Prediction and Application Centre (ICPAC), Nairobi, Kenya

**Keywords:** Climate sciences, Environmental sciences

## Abstract

Changes in wet and dry patterns have an impact on rain-fed agriculture, crop productivity, and food security in Eastern Africa. The purpose of this research is to look into the changes in wet days and dry periods within the Intergovernmental Authority on Development (IGAD) region. Climate Hazards Group Infrared Precipitation with Station Data (CHIRPS) and Multi Models Ensembles (MME) of 10 historical simulations and projections from Coupled Model Intercomparison Project (CMIP6) models were employed as the data source. Several statistical approaches, as well as wet and dry spell thresholds, were used to calculate patterns of change in wet and dry spells on a decadal (10-year), 20, 30, and 41-year time scale. The results show the region exhibits decrease a decrease in the number of wet days and protracted dry spells in the 1980s, followed by an extraordinary (exceptional) increase in wet days in the subsequent decades (2011–2020) during March–May (MAM), June–September (JJAS), and October-December (OND). In Kenya, Somalia, southeastern Ethiopia, Eritrea, and Djibouti, the probability of surpassing 7, 14, 21, 28 days (1, 2, 3, 4 spells) was less than 5%. Furthermore, floods in 1997, 2018, 2019, and 2020, as well as droughts in 1983, 1984, 1985, and 2021, were triggered by an increase or decrease in the number of wet days and dry spells over most of the region. The number of wet days is projected to decrease by 10–20% during the MAM season across Sudan, South Sudan, and central and northern Ethiopia, JJAS is projected to increase by 30–50% across central and northern Sudan. However, during the OND season, increases are projected over Uganda, Ethiopia, and Kenya under three Shared Socioeconomic Pathways (SSP1-2.6, SSP2-4.5, and SSP5-8.5) scenarios. These findings contributed to the advancement of scientific knowledge in the IGAD region, as well as decision-making, food security, and the development of adaptation and mitigation strategies. We encourage rain-fed agriculture, crop variety planning, and irrigation supplement.

## Introduction

Africa is one of the most vulnerable continents to climate change^1^ due to dependability on climate sensitive livelihoods and low adaptive capacity^2^. Rainfall is the most important meteorological variable for rain-fed agriculture production in East Africa^3^. The Gross domestic product (GDP) of most countries in Equatorial East Africa is sensitive to changes and variability in extreme events^4^. Agriculture plays a vital role in the lives of the people of East Africa, with around 80% of the population being engaged in agricultural activities, especially smallholder farming^5^. The observed trends in frequencies and intensities of dry spells pose a big threat to socio-economic sectors activities^6^, countries development agenda^7^, agriculture planning and main staple food production stability^7^, crop growth and food security^8^. Numerous studies around the world have noted an increase in dry season and dry spell lengths over North-Eastern North America^9^, China during 1960–2013^10^, Northern Tunisia^11^, Western and Central Sahel, Sudanian zone and Guinea Coast^12^. Thoithi^13^ reported an increase in wet days areas across Southern Africa between 1982–2019. Also, significant changes in the duration and timing of extreme dry and wet spells were reported over many parts of the United States, Europe and Australia^14^.

Rainy season over East Africa is highly variable in both space and time^15^, it begins in February over southern parts of Uganda to central and northern parts of Sudan in July and August. The long rains which occur between March and May (MAM) are crucial for sub-regions within equatorial East Africa^16^. The JJAS season accounts for 65–95% over Ethiopia^17^, 80–95% over South Sudan^18^ and 95–99% of total annual rainfall over Sudan^19^. The features of total rainfall, intensity, and variability are defined by wet and dry events daily^20^. Wet days are sequences of days with rain, conversely, dry spells are sequences of days without rain at 1 mm minimum threshold^21^. The intensity and duration of droughts and floods, and extra-ordinary heavy rainfall is directly proportional to the number of days with/without rainfall^22^. Wet and dry spells in West Africa appear to be closely linked to the spatio-temporal variability of the West African monsoon^12^.

Researchers use Climdex indices such as Consecutive Wet Days (CWD) and Consecutive Dry Days (CDD) on annual and seasonal time scales to assess patterns of extreme rainfall and seasonal drought over East Africa and beyond^22–26^. Furthermore, the potential impact of 1.5 °C and 2 °C Global Warming (GW) on CWD and CDD has been addressed widely. For example, the projected effects of 1.5 °C and 2 °C GW levels on the June–September season indicate an overwhelming likelihood of a reduction in CWD over most of GHA^27^. Some areas in West Africa such as the Guinea Coast, are projected to experience a decrease in CWD at both 1.5 C and 2 C WG. The length of a maximum dry period has been observed to be decreasing in April and May, and the probability of 8 consecutive days of dry spell is high (38–69%) in March, April, and August within Lake Kyoga Basin in Uganda^28^.

Excessive rainy days enhance the likelihood of floods, whereas excessive dry spells lead to droughts and their various impacts^29^.

Observed change and variability in wet days and dry spells determined the types of cash and food crops. Also, the activities such as where and when to plan, how to best prepare the agricultural land, and suitability of soil for crops^30^. The prolonged wet spells/dry spells could be referred to as floods and drought^31^. Long wet days/wet spells, and dry days/dry spells are of drought impacts indicators and pose severe risks to socioeconomic activities^32^. Putting into account climatic conditions of any region, the extreme wet spells can lead to saturated soils and thus influence the flood hazard^14^. Also, other related impacts on water- related vector-borne diseases^33^, water quality^34^, river flow and urban water management^35^. Regional, national and local information on wet days and dry spells are critical for monitoring and managing the current and future impacts and developing sustainable food security interventions^36^.

Many studies in eastern Africa have highlighted the importance of evaluating observed changes in wet and dry spells, heatwaves, droughts, flood patterns, and other unexpected, extraordinary extreme weather occurrences throughout main rainy seasons^4^. The prolonged wet days and dry spells combined with other socio-economic factors cause crop yield reduction, food insecurity, and water scarcity. These conditions normally force pastoralist communities to migrate to other regions in search of water and green pasture for their livestock. The information on the mean state of wet/dry days and wet/dry spell period is necessary in the determination of frequency, duration and intensity of the rainfall variability^37^. The duration of wet/dry days or wet/dry spells determines the supply and demand levels of the water resources^38^. Prolonged wet/dry days and wet/dry spells appear to be a significant factor for rain-fed food crops over Africa, Sahel^39^, Eastern Africa^40^. The characteristics of wet/dry spells in terms of frequency, duration and magnitude over equatorial East Africa revealed more wet spells with precipitation of above 14 mm per day^4^. research by^41^ found that 1-day wet and dry spells were dominant in western Kenya. The duration and magnitude of wet/dry spells varies with climatic zones and there was no clear monotonic trend in wet/dry spells over the Akobo Basin of Ethiopia^42^.

To the best of our knowledge, there are still some limitations regarding the probability of wet days and dry spells to exceed well defined thresholds such as the probability of exceeding 7, 14, 21 and 28 consecutive wet days (1, 2, 3, 4 spell) at the regional level which are critical for regional rain-fed agriculture and irrigation planning among others. Therefore, the main objective of this paper is to fill the information gaps on observed and projected changes in wet days and dry spells for three peak rainfall seasons (MAM, JJAS, OND) and DJF season over the IGAD region of Eastern Africa.

## Data and methods

### Study area

**Figure 1:**
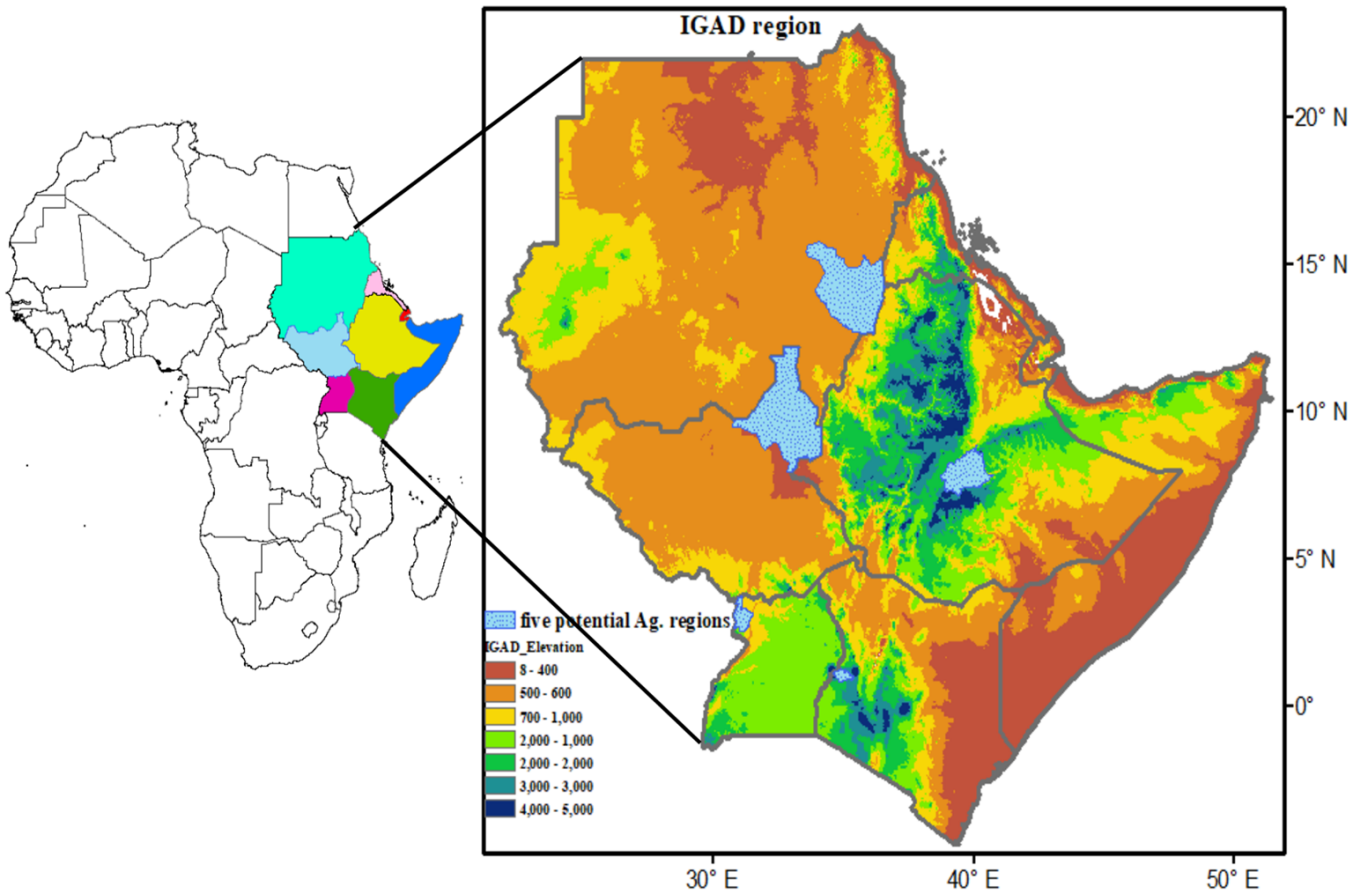
Elevation map of the IGAD region of Eastern Africa, the sky blue sub-regions are five potential agricultural areas (El Gadaref state in Sudan, Upper Nile state in South Sudan, Arsi zone in Ethiopia, Trans Nzoia county in Kenya, Arua district in Uganda) used in validation inter-annual variability of wet days and dry spells. The data used are NASA -Digital Elevation Model (DEM) from Space Shuttle Radar Topography Mission (SRTM).

The study focuses on Intergovernmental Authority on Development (IGAD) member states of Sudan, Eritrea, Djibouti, South Sudan, Ethiopia, Kenya, Somalia and Uganda (Fig. 1). The geographical coordinates of the region are latitude 21.4°–51.2° E and Longitude 5°–23.2° N. The region is characterized by complex topography that varies from an area below sea level over Sudan to the highest points of Mount Kenya at 5199 m as the second highest mountain in Africa after Mount Kilimanjaro (5895 m) in Tanzania, both Rift Valley in Kenya and Ethiopia.

The IGAD region climate is affected severely by these high elevation landmarks, and seasonal movement of the intertropical convergence zone (ITCZ) north and southward. The ITCZ is the one of factors that determine the variation in four different rainfall seasons such as December, January, February (DJF), March, April, May (MAM), June, July, August, September (JJAS) and October, November, December (OND). Also, many studies show the climate of the region is influenced by El Nino/Southern Oscillation^43–46^ as well as variability of sea-surface temperature over the Indian Ocean^47^. The different ENSO phases (El Niño and La Niña or neutral) have different impacts over different parts of the region^46,48^. The variation in climatic zones whether warm deserts or humid highland climates are mainly driven by orography, geography and micro- synoptic systems^49^. This situation offers an opportunity or could be a challenge affecting the accuracy of satellite rainfall estimates over the region. Due to the importance of wet days and dry spells for rain-fed agriculture, we selected five areas in the region considered as a food basket for the IGAD region. These areas are labelled with sky blue color (El Gadaref state in Sudan, Upper Nile state in South Sudan, Arsi zone in Ethiopia, Trans Nzoia county in Kenya, Arua district in Uganda) used to access the temporal patterns of wet and dry spells over the region.

### Datasets

Climate Hazards Group Infrared Precipitation with in-situ station (CHIRPS) datasets from the University of California at Santa Barbara (UCSB) were selected for this study. CHIRPS product is a high-resolution Satellite Rainfall Estimate (SRE) blended with in-situ station data at 0.05° spatial resolution at daily, pentad, dekadal, and monthly temporal resolution^24^. In this study, we used the daily datasets from 1981 to 2021. The multiple steps detailed major input datasets and processes, merging with station data, weighted bias ratios, homogeneity of the time series and algorithm used to generate CHIRPS products provided in^24,50^. Other satellite products derived from in-situ observation such as TAMSAT v3.1, PERSIANN-CDR, CPC ARC2, CPC RFEv2, TRMM 3B42RT v7, CPC V1.0, CMORPH v1.0 CRT, IMERG v06 daily data used to complement CHIRPS v2.0 daily data. This study also employs Multi Models Ensembles (MME) of 10 daily historical simulations and projections from CMIP6 models^51^ for three different emission scenarios: a low (Shared Socioeconomic Pathway (SSP) 126 scenario), medium (SSP 245 scenario) and high (SSP585 scenario). Table 1 contains a complete list of the models that were utilized. These 10 models were chosen based on how well they performed across the IGAD region^52^. The selected 10 models and CHIRPS datasets were rescaled from original resolutions to ten-kilometer (0.1 deg) using the bilinear interpolation method^53^ to overcome the challenges of differences in resolutions of CMIP6 models and CHRIPS satellite rainfall estimates. The full model description including the spatial resolution, member realization outputs, the institute(s) possessing the intellectual property rights, and the full description of abbreviation of the names and datasets used can be found in CMIP6 institution_id values (http://wcrp-cmip.github.io) website.

**Table 1:** List of 10 CMIP6 models used in this study and their institutions, model names and spatial resolutions.

	CMIP6 model name	Institution	Country	Coarse horizontal resolution
1	BCC-CSM2-MR	BBC	China	1.1° × 1.1°
2	CMCC-CM2-HR4	CMCC	Italy	0.942° × 1.25°
3	EC-Earth3	EC-Earth Consortium	Europe	0.7° × 0.7°
4	GFDL-ESM4	NOAA-GFDL	USA	1.3° × 1°
5	HadGEM3-GC31-MM	MOHC	UK	0.942° × 1.25°
6	INM-CM5-0	INM	Russia	2° × 1.5°
7	IPSL-CM6A-LR	IPSL	France	2.5° × 1.3°
8	MIROC6	JAMSTEC	Japan	1.4° × 1.4°
9	NorESM2-MM	NCC	Norway	0.94° × 1.25°
10	TaiESM1	CcliCS	Taiwan	1.25◦ × 0.94◦

### Methods

#### Criterion and threshold for Wet days and dry spells

There are a considerable number of definitions and thresholds for calculating wet days and dry spell patterns in the Literature. In most cases, these definitions and thresholds produce different wet/dry days and spells even when applied to the same observation or gridded dataset^27^. The first and most used methods in the literature are those that apply threshold values on Total rainfall amount^17,54–57^, Fraction of evapotranspiration^58^, Walter method^59^, Number of rainy days and spell lengths^27^. All these methods have limitations regarding a number of rainy days within the threshold of total rainfall amount, seasonal extremes indices and probability of exceeding defined wet days and dry spells. Therefore, in this study wet days and dry spells criterion and thresholds (Table 2) were determined by adopting a threshold of 1 mm per day used to define a wet/dry day, 7 consecutive wet days (1 wet spell), 7 consecutive dry spells (1 dry spells), probability of exceeding 7, 14, 21 and 28 wet days (1, 2, 3, 4 spells). The adoption of these criteria is informed by the close relationship between wet days, dry spells and agricultural applications. Also, the type of rainfall necessary for the development of food crops such as maize and sorghum in various agricultural locations with varying climatic zones. In addition, adopting a threshold of 1 mm to avoid unreasonable wet days and dry spells may arise from applying the World Meteorological Organization's (WMO) recommended rainy day threshold of 0.1 mm for studies using in-situ observations. In addition, the selection of rainfall thresholds in this study is informed by1mm threshold used in Climdex indices to define the maximum number of Consecutive Wet Days **(**CWD) and a maximum number of Consecutive Dry Days** (**CDD). The indices are used by many researchers around the world on an annual time scale^22–26^.

**Table 2:** Criterion and threshold for wet/dry days and spells.

Wet/dry days and spells criterion and threshold will be based on rainy days and spell lengths
Threshold for wet days: ≥ 1 mm
Threshold for dry days: ≤ 1 mm
Threshold for wet spells:7 consecutive days at least with 1 mm rain
Threshold for dry spells: 7 consecutive days at least without 1 mm rain
Probability of exceeding for wet days: 7, 14, 21 and 28 wet days
Probability of exceeding for dry spells: 1, 2, 3 and 4 dry spells
For MAM season: 92 days are maximum number of wet days or 13 dry spells
For JJAS season: 122 days are maximum number of wet days or 17 dry spells
For OND season: 91 days are maximum number of wet days or 13 dry spells
For DJF season: 90 days are maximum number of wet days or 12 dry spells

#### Statistical methods for wet days and dry spells

Before computing wet days and dry spell patterns using CHIRPS products, the other satellite-derived products from in-situ were utilized to assist comparison before computing wet days and dry spells patterns using CHIRPS products. TAMSAT v3.1, PERSIANN-CDR, CPC ARC2, CPCRFEv2, TRMM 3B42RT v7, CPC v1.0, CMORPH v1.0 CRT, and IMERG v06 are the products that were used. Due to differences in these datasets' resolutions, a bilinear interpolation method was used to rescale (or "regrid") all of the data to 0.1 degrees (10 km). The data sets from 2001 to 2020 were used to compute the spatial patterns of wet days and dry spells over the IGAD region.

Mean wet days and dry spells were calculated using an arithmetic mean over decadal (10, 20, 30, and 41 years). The climatology period of 30 years (1981–2010) has been used as the baseline for anomaly detection. To compute future changes in wet days and dry spells, we selected 1985–2014 as the base period, along with two future time frames, referred to hereafter as near-future (2021–2050) and far-future (2071–2100). The arithmetic mean for wet and dry spells is the sum of the number of days with at least a 1 mm threshold divided by the number of years included in the analysis, as described in Eq. (1).

1 $$\overline{X }=\frac{1}{{\varvec{N}}}\sum_{{\varvec{i}}=1}^{{\varvec{n}}}{{\varvec{X}}}_{{\varvec{i}}}$$

where $\overline{X }$ = average number of wet days and dry spells, n is the number of years sample, which are 10, 20, 30 and 41 years, ${X}_{i}$ = the value of each season and yearly number of wet or dry spells being averaged. For the purpose of this study, the means computed on decadal are 1981–1990 (1980s), 1991–2000 (1990s), 2001–2010 (2000s), 2011–2020 (2010s), 20 years (1981–2000 and 2001–2020), 30 year or climatology (1981–2010) and 41 years (1981–2022) to assess the mean state of wet days and dry spells.

We computed the Probability of exceedance, which is a statistical metric describing the probability of the accumulated number of wet days will be met or exceeded at 1 mm/day as wet day thresholds and 7, 14, 21 and 28 consecutive (continuous) days as wet spells thresholds. The Probability of exceedance counted based on 7, 14, 21, 28 consecutive wet days (1, 2, 3, 4 wet spells) following the start of the first season for MAM (61 days), JJAS (122 days), OND (92 days) and DJF (90 days) for 1981–2010 climatology period.

The changes ($\nabla \mathbf{X}$) in wet and dry spells on decadal (1980s, 1990s, 2000s, 2010s) and difference in two means of current 20 years (2001–2020) compared with the previous 20 years (1981–2000) used to assess whether there is shift in mean state of wet days and dry spells (increased or decreased) and dry spells (prolonged dry spells). It can be written as in Eq. (2) below.

2 $$\nabla \mathbf{X}=\frac{1}{{\varvec{N}}}(\sum_{{\varvec{j}}=1}^{{\varvec{n}}}{{\varvec{X}}}_{{\varvec{j}}})-(\frac{1}{{\varvec{N}}}\sum_{{\varvec{i}}=1}^{{\varvec{n}}}{{\varvec{X}}}_{{\varvec{i}}})$$

where, (${{\varvec{X}}}_{{\varvec{j}}}$) represent 1980s,1990s,2000s,2010s and current 20 years (2001–2020) and represented previous 20 years (1981–2000), (${{\varvec{X}}}_{{\varvec{i}}}$) represent climatology period (1981–2010) as baseline to measure the changes in wet days and dry spells. Then the rate of change in percentage (%) computed using Eq. (3).

3 $$\mathbf{Rate \,of \,change}\left(\nabla \mathbf{X}{\%}\right)=\left(\frac{\nabla \mathbf{X}}{{{\varvec{X}}}_{{\varvec{i}}}}\right)\boldsymbol{*}100$$

To assess the implication of wet days and dry spell on drought and floods, the standardized anomaly of wet and dry spells patterns for five potential agricultural areas was computed to facilitate comparison with the Standardized Precipitation Index (SPI). This method is widely used to objectively rank synoptic-scale events^60,61^. This study examined the temporal patterns of wet days’ anomalies from 1981 to 2022 for each of the three seasons (MAM, JJAS, and OND). Open-source statistical R-Package Climate Data Tool (CDT v7.0))^62^, Climate Data Operators (CDO) command line operators and ArcMap10.4 used to create the maps (ArcGIS for Desktop 10.4 Download—arcview.exe (informer.com)).

### Consent to participate

All authors consent to participate.

## Results

### Mean wet days and dry spells

**Figure 2:**
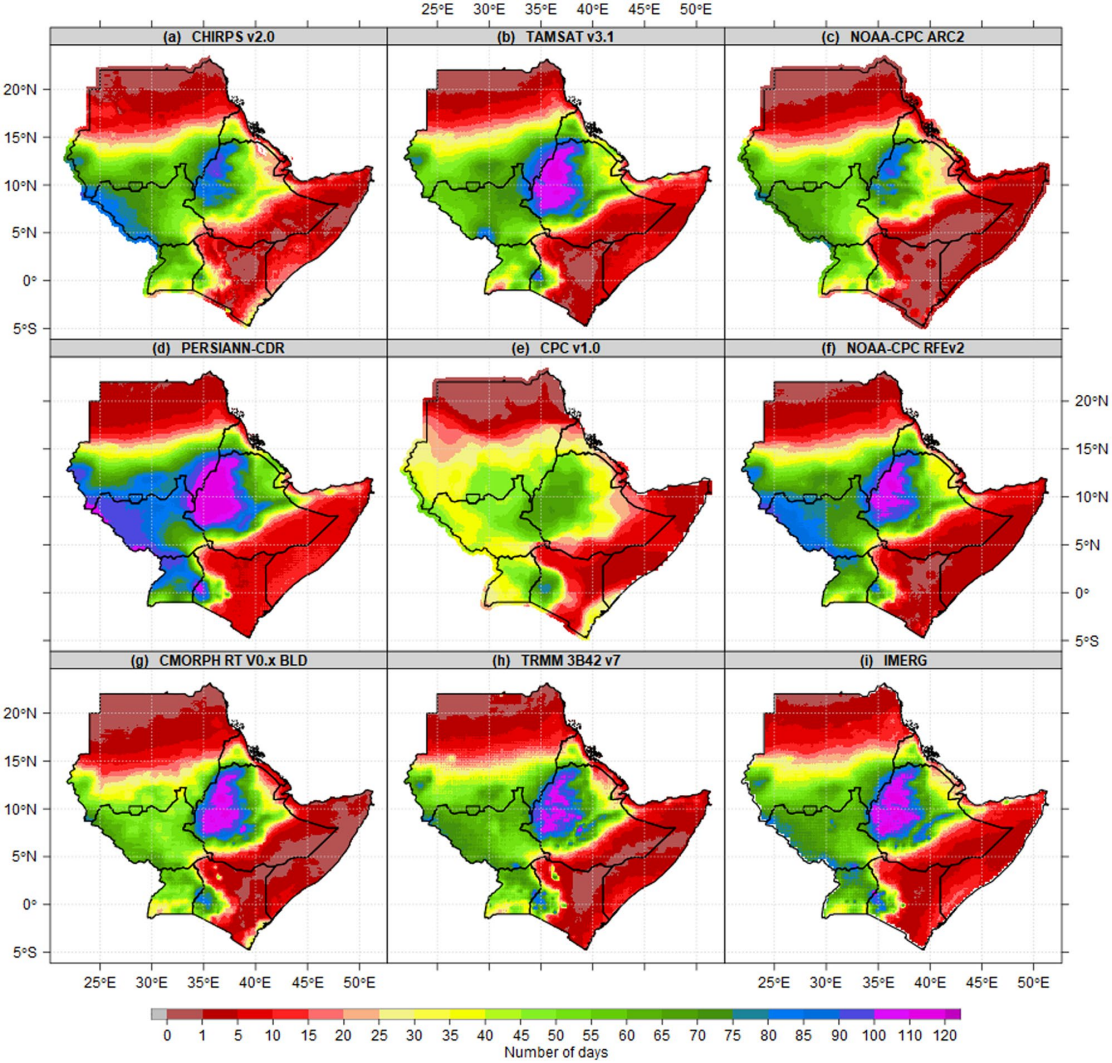
Comparison of JJAS means state of wet days of 9 gridded satellite datasets derived from the in-situ seasonal mean state of Number of wet days patterns(days/season) at 1 mm thresholds for the period 1981–2010 reference period. The pixel values in the legend are presented as the number of wet days.

To facilitate the comparison of CHIRPS daily data with other 8 gridded Satellites derived from in-situ during the JJAS season in the IGAD region of eastern Africa used as results sample. The results show the highest number of wet days was recorded over the highlands of western Ethiopia and the western part of South Sudan (Fig. 2). These patterns of the highest wet days may explain the patterns of rainfall onset and cessation^63^ and the highest total rainfall amounts observed over South Sudan^18^, Kiremt rainy season over Ethiopia^17^ and Sudan^64^ during the JJAS season. More than 100 wets days were recorded over the highlands of western Ethiopia based on TAMSAT v3.1, NOAA-CPC RFEv2, PERSIANN-CDR, CMORPH RT V0.x BLD, TRMM 3B42 v7 and CPC v1.0. Out of 122 possible wet days during JJAS, the ASALs in Kenya, Somalia, southeastern Ethiopia and northern parts of Sudan recorded less than 15 wet days for all 9 gridded products. The CPC v1.0 has fewer wet days over Uganda, South Sudan and Ethiopia compared to other products. Even though JJAS is the dry season in most parts of Kenya, the highlands of western and Nyanza counties had more than 50 wet days. This indicates a high likelihood of success for rain-fed agricultural activities throughout the year considering the patterns of MAM and OND as the peak of the Long Rains and Short Rains over Kenya^15^.

**Figure 3:**
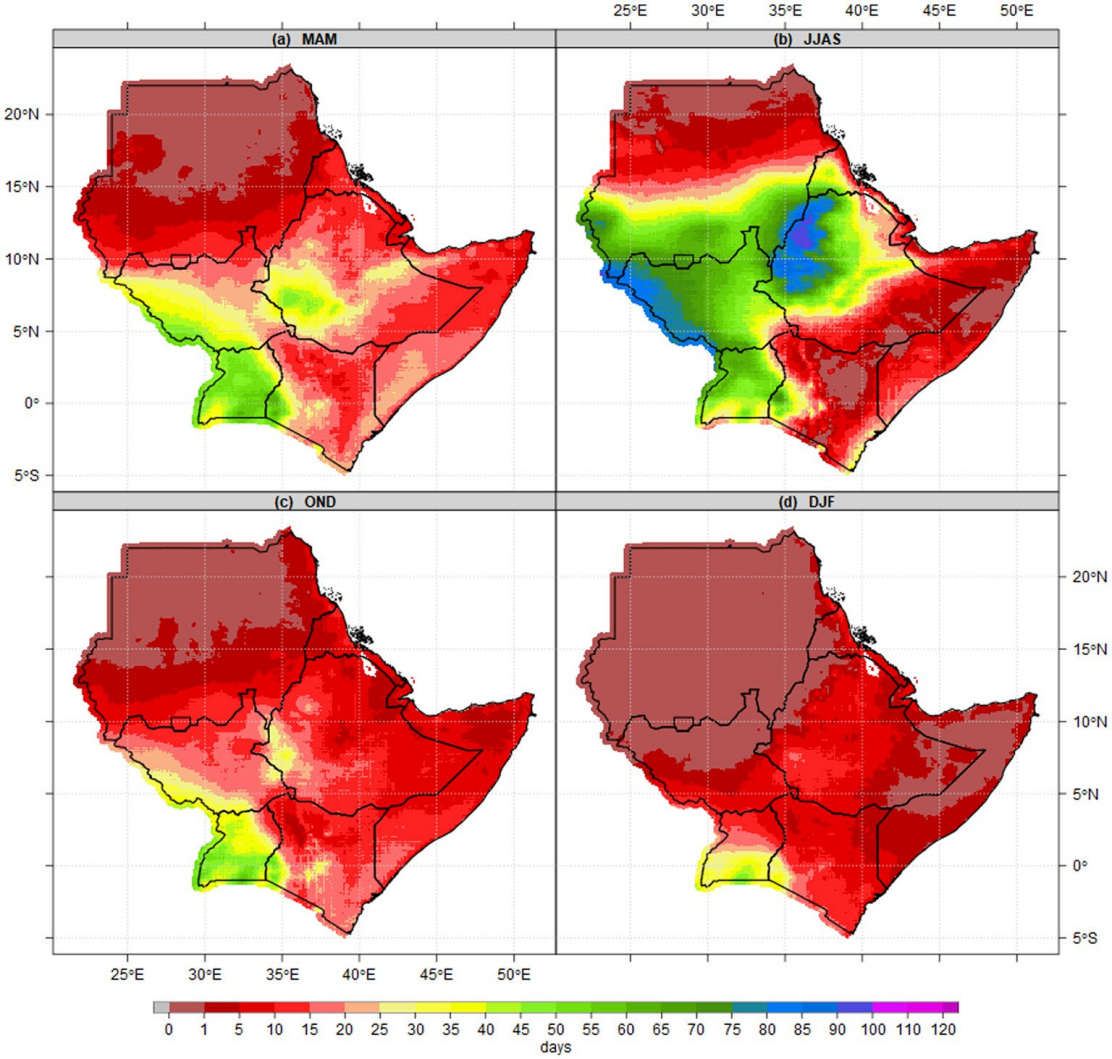
Seasonal mean state of Number of wet days patterns(days/season) at 1 mm thresholds for the period 1981–2010 reference period during (**a**) MAM, (**b**) JJAS, (**c**) OND and (**d**) DJF. The pixel values in the legend are presented as a number of wet days.

Figure 3 shows the average number of wet days during MAM, JJAS, OND and DJF seasons based on CHIRPS datasets. Generally, the results show the areas with the highest number of wet days vary from season to season and within countries. On a seasonal timescale, the MAM season, the highest number of wet days (55–65 days) are recorded over southwestern South Sudan, southern and south-Eastern parts of Uganda, Lake Victoria basin and the highland of western and Nyanza region of Kenya. Findings by[41]found that the seasonal rainfall totals and the number of wet days at the sub-regional level in Equatorial Eastern Africa had the highest level of spatial coherence. The Majority of districts in Sudan, Eritrea, Djibouti, northern Somalia, Upper Nile state in South Sudan, southeastern, northern and northeastern Ethiopia, and northeastern Kenya recorded the lowest number of wet days (Fig. 3a). Similarly, the highest number of wet days (80–100) during JJAS were recorded over western South Sudan, the highlands of western Ethiopia, while northern Sudan, southeastern Ethiopia zones, the majority of counties in Kenya, most parts of districts in Somalia and coastal Djibouti recorded the lowest number of wet days (Fig. 3b). In OND season, the highest number of wet days were recorded over most parts of Uganda and Nyanza counties in Kenya. Most parts of Sudan, eastern and southeastern Ethiopia, Kenya, Somalia, Djibouti, and Eritrea recorded the lowest number of wet days ranging between 45 and 60 dry days (Fig. 3c). During the DJF season, with the exception of southern and central Uganda, Lake Victoria basin, the rest of IGAD regions recorded the lowest wet days ranging between 0 and 10 dry days (Fig. 3d).

**Figure 4:**
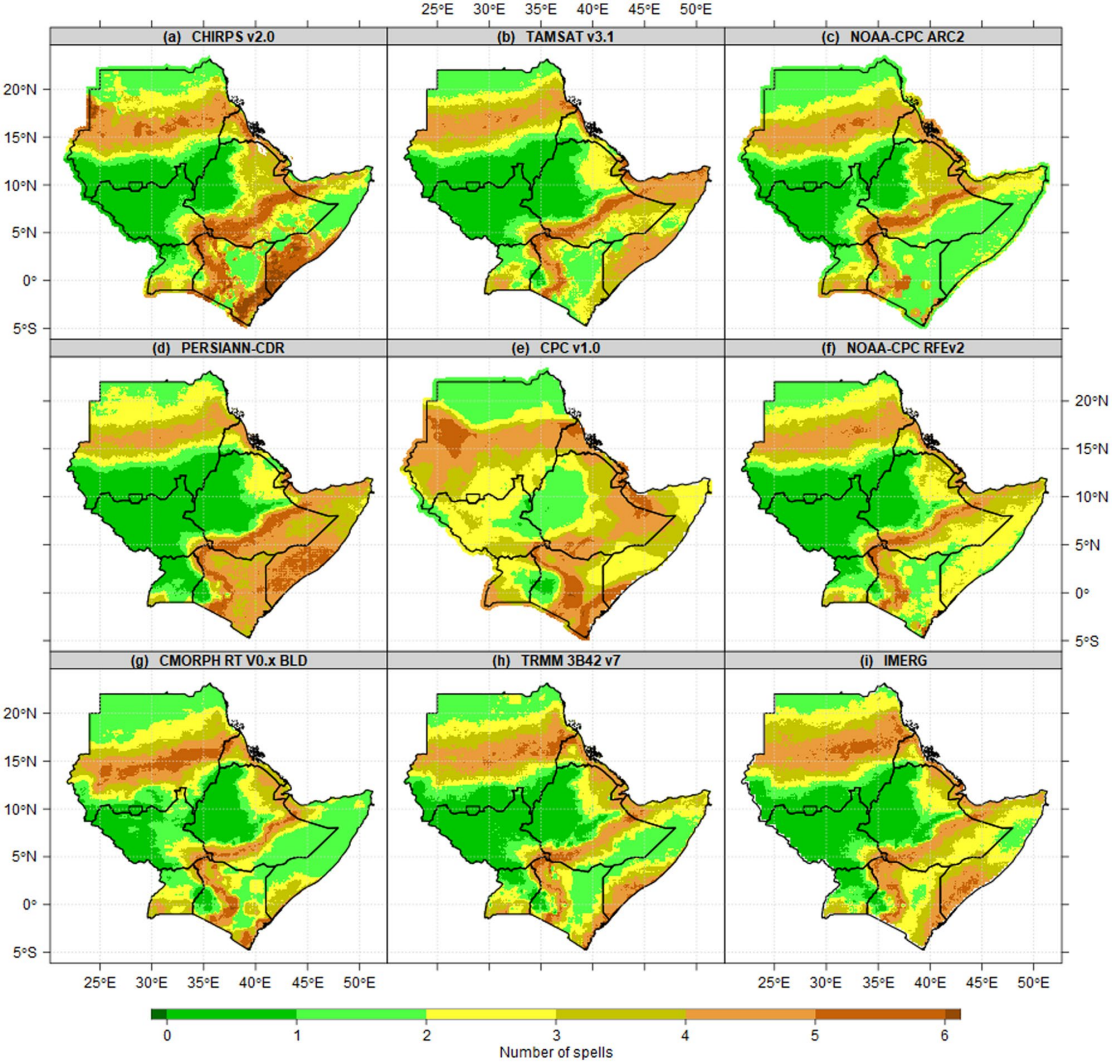
Comparison of JJAS mean state of wet days of 9 gridded Satellite derived from in-situ seasonal mean state of Number of dry spells patterns(days/season) at 1 mm thresholds for the period 1981–2010 reference period. The pixel values in the legend are presented as a number of dry spells.

Figure 4 presents the comparison of mean dry spells(climatology) patterns of 9 gridded satellites derived from in-situ during JJAS seasons. Except CPC v1.0, other datasets agreed on 0–1 spells as the shortest period, which was observed over southwestern South Sudan, the highlands of central and western Ethiopia. The longest continued dry-spells recorded over ASALs in Kenya, Somalia, southeastern Kenya, Djibouti, Eritrea and squalled line in central Sudan.

**Figure 5:**
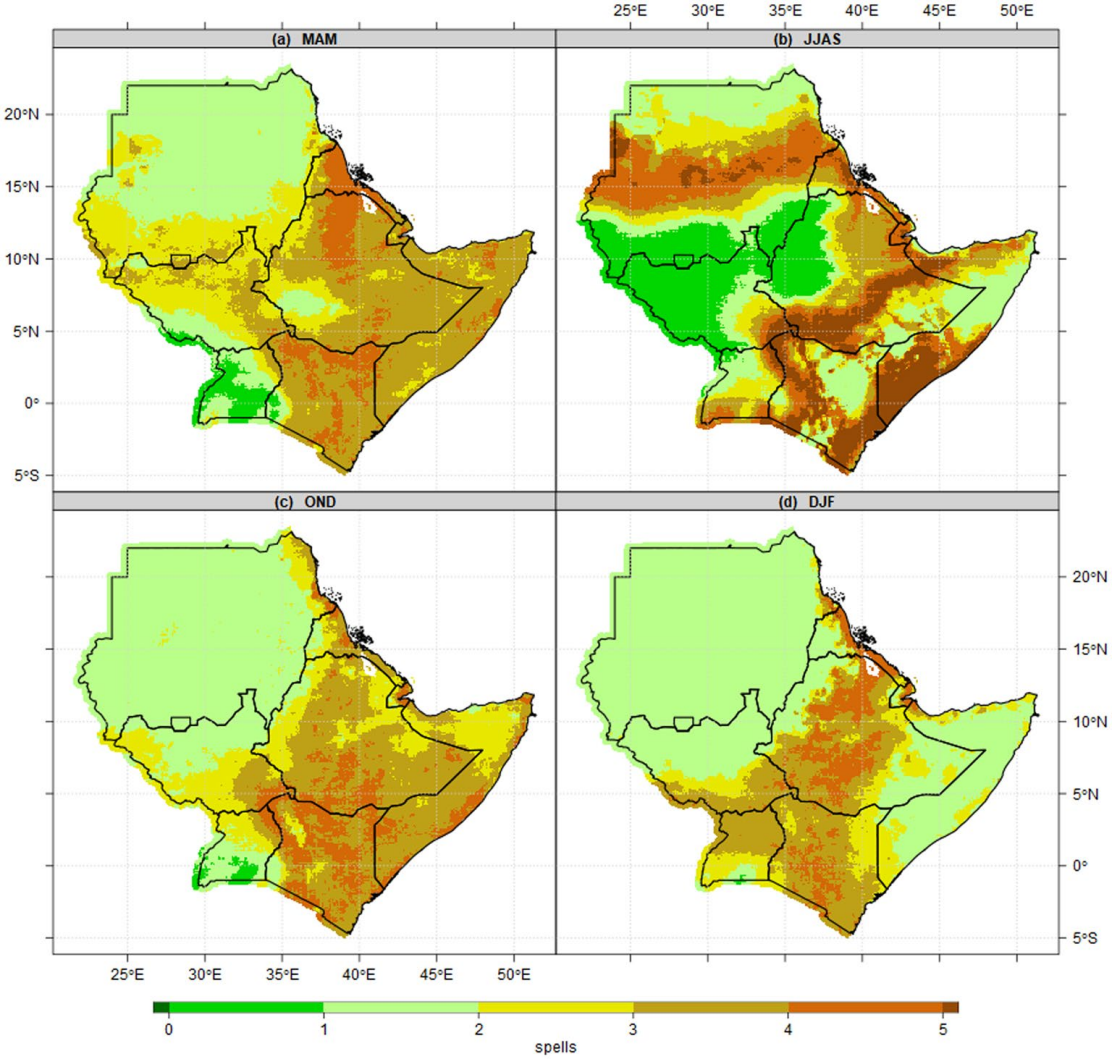
Seasonal mean state of Number of wet days patterns(days/season) at 1 mm thresholds for the period 1981–2010 reference period during (**a**) MAM, (**b**) JJAS, (**c**) OND and (**d**) DJF. The pixel values in the legend are presented as numbers of dry spells.

Figure 5 presents the mean dry spells(climatology) patterns during four seasons. Conversely, the shortest and longest continued dry-spells recorded over areas with the highest and lowest wet days as described in Fig. 4 above. Most parts of Kenya, Somalia and Ethiopia recorded 3–5 consecutive dry spells (21–35 consecutive dry days) during MAM (Fig. 5a). The southwestern and northern parts of South Sudan, the highland of western Ethiopia, and rain belts in southern parts of Sudan recorded less than 1 spell as the shortest spell in the IGAD region during the JJAS season (Fig. 5b). Nevertheless, OND season shows 3–5 spells (21–35 consecutive dry days) recorded over most parts of Kenya, Somalia and southeastern Ethiopia. The majority of districts in southwestern Uganda recorded 1 spell as the longest (Fig. 5c). In a related study, an increase in the length of maximum dry spells was reported across the majority of locations in April and May over Uganda's Lake Kyoga Basin^28^. Similarly, central and northern Uganda, most parts of Kenya, northeastern, central and southwestern Ethiopia have the longest consecutive spells during DJF season (Fig. 5d). Lake Victoria basin consistently observed lowest the spells across all four seasons.

**Figure 6:**
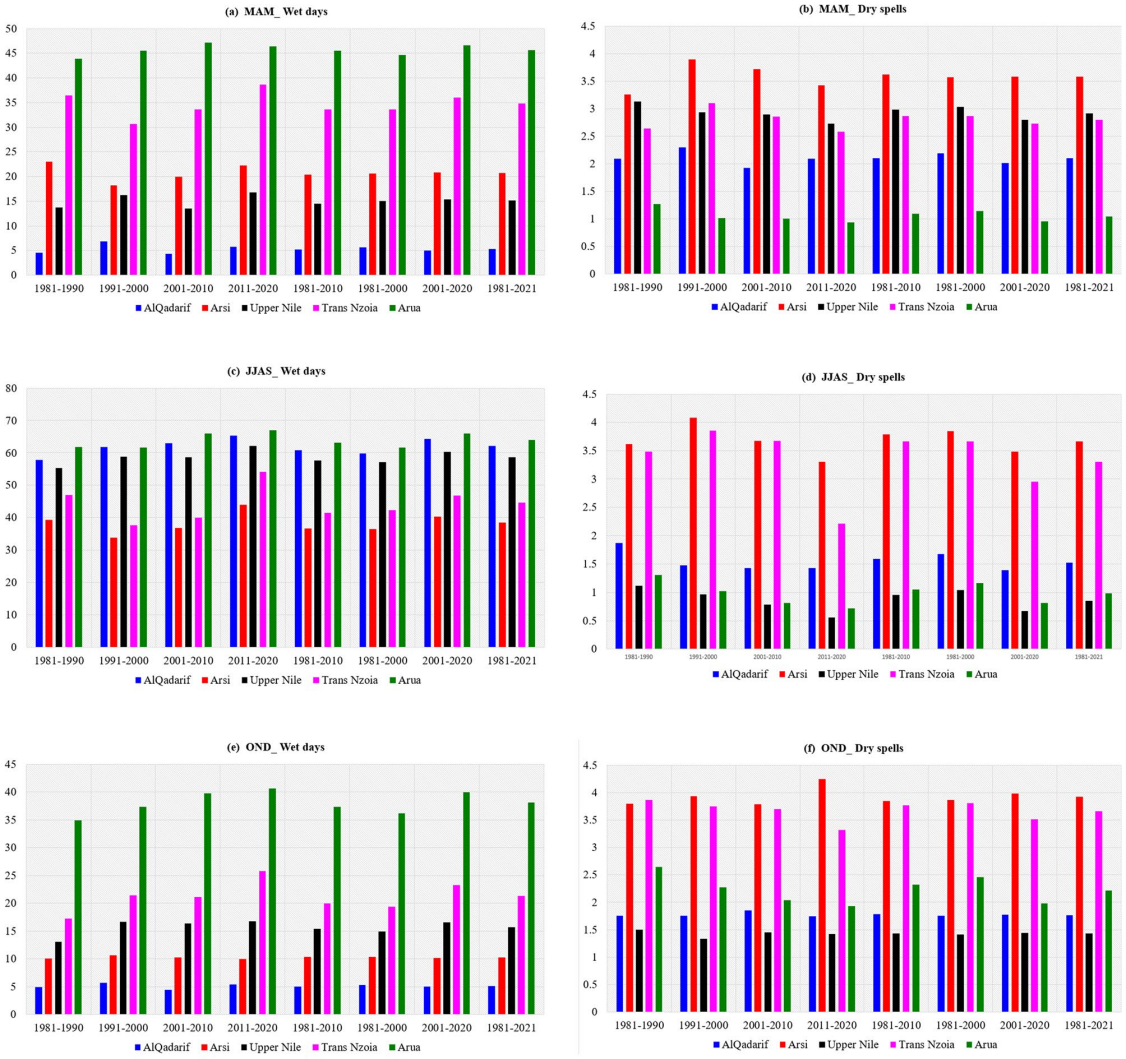
Histogram illustrate wet days (first column) and dry spells (second column) means in last 41 years (1981–2021), four decades (1981–1990, 1991–2000, 2001–2010 and 2011–2020), current 20 year (2001–2020) and previous 20 years (1981–2000) over El Gadaref in Sudan, El renk in South Sudan, Arsi in Ethiopia, Trans Nzoia in Kenya, Arua in Uganda.

The variation in an average number of wet days and dry spells over Arua, AlQadarif, Upper Nile, Arsi, and Trans Nzoia is presented in Fig. 6. The variation assessed during the 1980s, 1990s, 2000s, and 2010s (1981–1990, 1991–2000, 2001–2010, 2011–2020), 30 years’ average (1981–2010), 41 years’ average (1981–2021) and 20 years’ average (1981–2000, 2001–2020). The results revealed that the mean values for four decades (1980s, 1990s, 2000s, 2010s) and 41 years average (1981–2021) for MAM, JJAS and OND Seasons recorded very close mean values across all five regions. The different timescale means values for the MAM season showed that Arua in Uganda recorded the highest mean of wet days (lowest mean of consecutive dry spells). AlQadarif in Sudan recorded the lowest mean wet days (highest mean dry spells) during the season (Fig. 6a,b). The highest wet days (lowest dry spells) during JJAS were recorded over Arua and Upper Nile in South Sudan (Fig. 6c,d). The Arua recorded the highest mean of wet days (lowest mean consecutive dry spells) during JJAS and continued to OND (Fig. 6e,f). in Addition, the Arsi zone in Ethiopia recorded the lowest mean values of wet days (highest mean value of consecutive dry spells) during OND. The fluctuation of the Madden Julian Oscillation during the MAM and OND seasons has been connected to consecutive wet days and dry spells periods over most parts of East Africa^65^.

### Probability of wet days and dry spells exceeding defined thresholds

**Figure 7:**
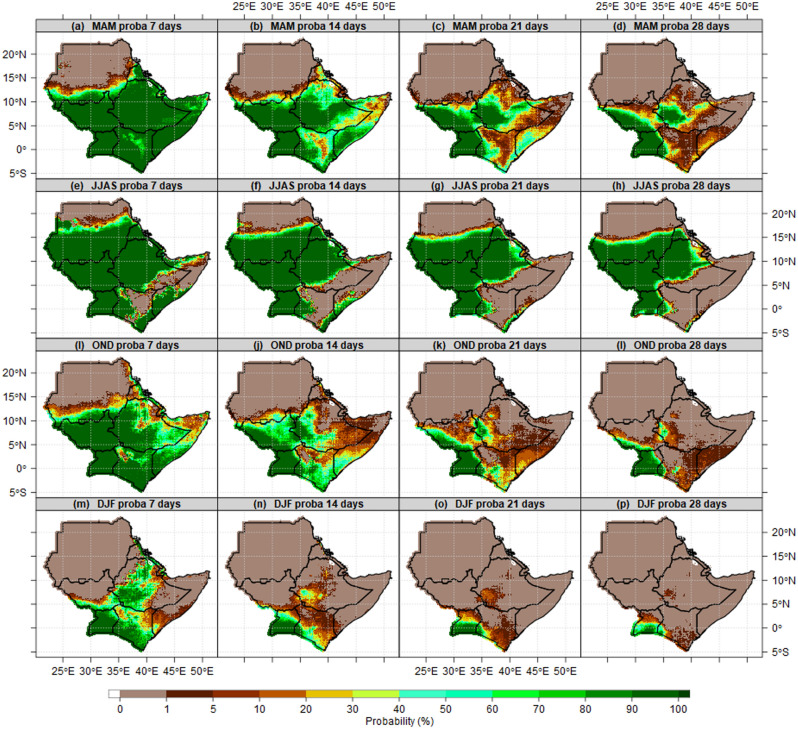
Probability of exceeding 7, 14, 21, 28 consecutive wet days at 1 mm rainfall thresholds during MAM (first raw), JJAS (second raw), OND (third raw) and DJF (fourth raw) season reference to 1981–2010 mean. For the period 1981–2010 reference period.

Figure 7 shows the probability of wet days exceeding 7, 14, 21, 28 consecutive wet days during MAM, JJAS, OND and DJF seasons. The results for the MAM season show that Uganda, South Sudan and Ethiopia, Kenya, Somalia, Djibouti and most parts of Eritrea observed more than 90% probability of wet days exceeding 7 consecutive wet days (Fig. 7a). However, the probability started shrinking continuously when 14, 21 and 28 consecutive wet days were examined. Southwestern South Sudan and Ethiopia, Uganda, and Nyanza countries in Kenya maintained more than 80% probability of exceeding 7, 14, 21 and 28 consecutive wet days during MAM (Fig. 7a–d) and JJAS seasons (Fig. 7e–h). Similarly, Lupi reported that in the Melkassa location in Central Rift Valley of Oromia State, Ethiopia, the likelihood of getting dry spells lasting 5, 7, and 10 days is less than 50%, and it lowers to under 20% at the start of the JJA peak season^66^. ASAL of Sudan, Kenya and Somalia recorded less than 1% probability of wet days exceeding 14, 21, and 28 consecutive days. Most parts of Uganda, South Sudan, Lake Victoria basin recorded more than 80% of wet days to exceed 14, 21, and 28 consecutive days across MAM, JJAS and OND season (Fig. 7i–l). Most parts of the IGAD region recorded less than 1% probability of wet days to exceed 14, 21, and 28 consecutive days during the DJF season (Fig. 7m–p). No changes in the pattern of the probability of exceeding 7–28 days over northern parts of Sudan across all four seasons (MAM, JJAS, OND and DJF) because these areas are dry climatologically.

**Figure 8:**
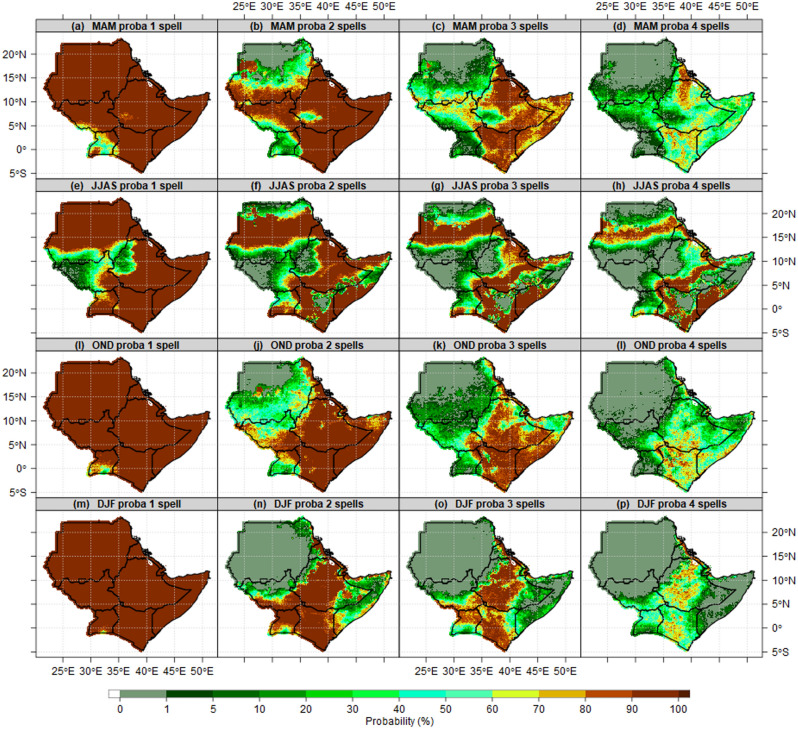
Probability of exceeding 1, 2, 3, 4 consecutive spells during at 1 mm rainfall thresholds during MAM (first raw), JJAS (second raw), OND (third raw) and DJF (fourth raw) season reference to 1981–2010 mean. For the period 1981–2010 reference period.

On the other hand, the probability of dry spells exceeds 1, 2, 3, 4 consecutive spells (7, 14, 21 and 28 days without rain) during MAM, JJAS, OND and DJF season presented in Fig. 8. The results show, the areas with lowest probability consecutive wet days (less than a 1%) discussed in Fig. 7 above recorded the highest (100%) probability of exceeding 1, 2, 3 and 4 consecutive dry spells (7, 14, 21, 28 days without rain) during MAM, JJAS, OND and DJF seasons (Fig. 8a–q). The patterns of dry spells in MAM over Eastern Uganda(70–90%) are different from the findings by Ojara et al., who reported that the probability of 8 days dry spell is high (38–69%) across all 9 weather stations over Lake Kyoga Basin in Uganda during in March, April, and August^28^. The difference is due to the usage of 7 and 8 days, and CHIRPS data appears to overestimate dry days compared to station data. Sudan, Eritrea, Ethiopia, Kenya, Djibouti and Somalia recorded a 100% probability of exceeding 1 consecutive dry spell (7 days without rain) during MAM, OND and DJF seasons (Fig. 8a,i,m). The patterns over Western South Sudan, southern parts of Sudan, and highlands of western Ethiopia are the only areas that recorded the lowest probability (less than 5%) of the probability of exceeding1 consecutive dry spell during JJAS season (Fig. 8e). Our finding is partially in agreement with study by^67^, who found the probability of three-dekad dry spells(30 days) was high at all stations in the Upper Awash River Basin, Ethiopia during the short rainy (Belg) season.

### Observed change in wet days and dry spells patterns

**Figure 9:**
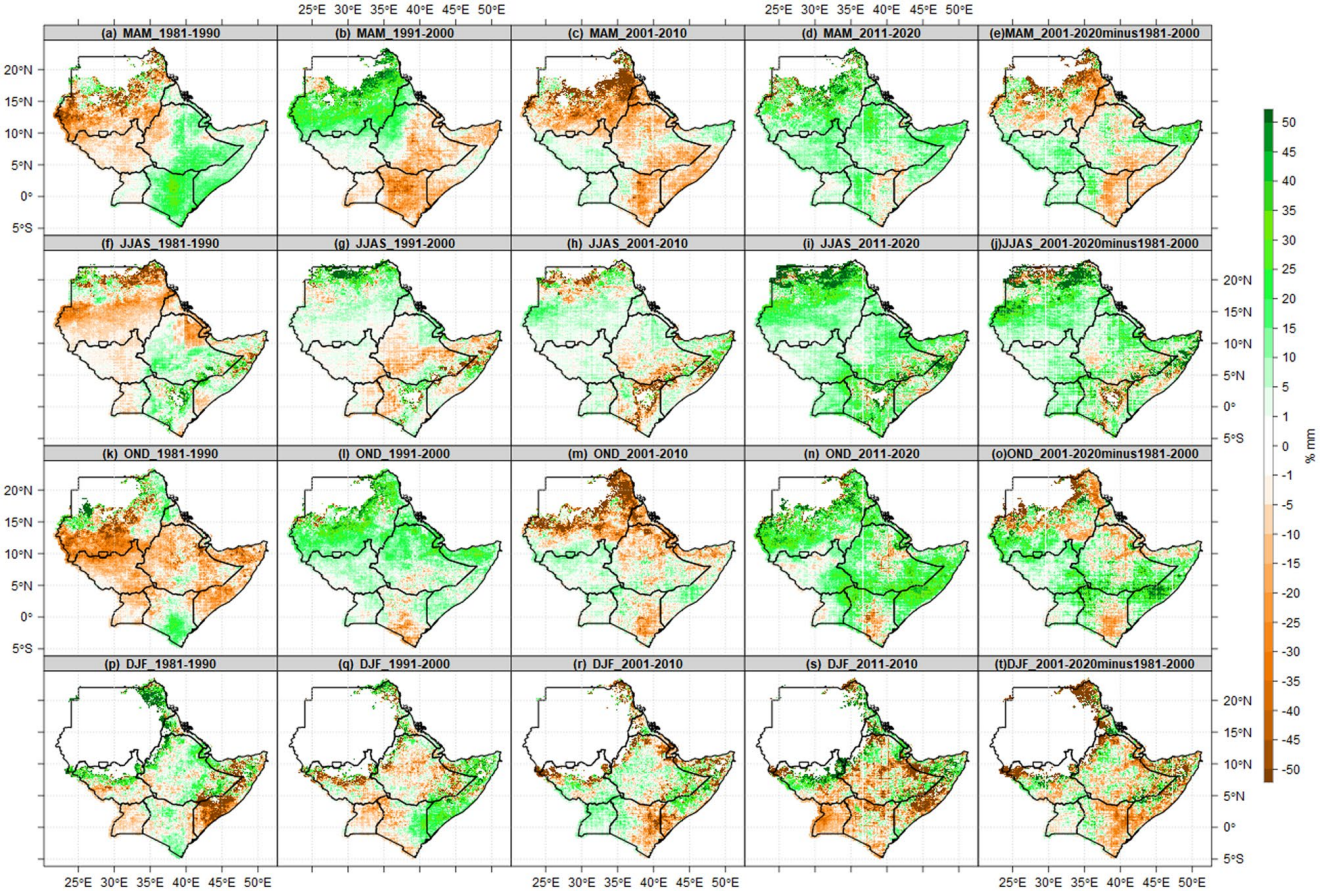
Changes in seasonal mean state of number of wet days at 1 mm thresholds for the four decades (1981–1990, 1991–2000, 2001–2010 and 2011–2020), average of 40 years (1981–2020) and differences between current 20 year (2001–2020) and previous 20 years (1981–2000). The Brown color (negative values) indicates decreased wet days dry, while the green color (positive values) indicates increased wet days.

Figure 9 presents the observed rate of change (%) in the seasonal mean state of the number of wet days at 1 mm thresholds for the four decades 1980s, 1990s, 2000s, and 2010s. The results show a considerable decadal (10-year) fluctuation in wet days across the IGAD region. During the recent decade (2011–2020), nearly all parts of the region experienced an exceptional (unusual) increased number of wet days during the MAM, JJAS, and OND seasons. In the 1980s, wet days increased by more than 35% throughout Kenya, southern parts of Somalia, southeast Ethiopia, western Ethiopia, and southern portions of Sudan. In the southern regions of Sudan during the JJAS season in the 1980s and 1990s, there was a 15–30% increase in rainy days in the 2010s. Similarly, during the OND season in the 1990s, 2000s, and 2010, most parts of South Sudan experienced a 5–15% increase in wet days. During the MAM season, there was a 15–35% decrease in the number of wet days across South Sudan in the 1980s, and throughout Kenya, Uganda, Somalia, and southeastern Ethiopia in the 1990s. Again, during all four seasons in the 1980s, wet days decreased by 15–35% over Sudan and South Sudan. In the 1990s, the JJAS season experienced less than a 10% decrease in wet days in most of Ethiopia and the Lake Victoria basin. The DJF season experienced a 5–15% decrease in the number of wet days in Uganda over the 1980s, 1990s, and 2010s (Fig. 9p–t).

**Figure 10:**
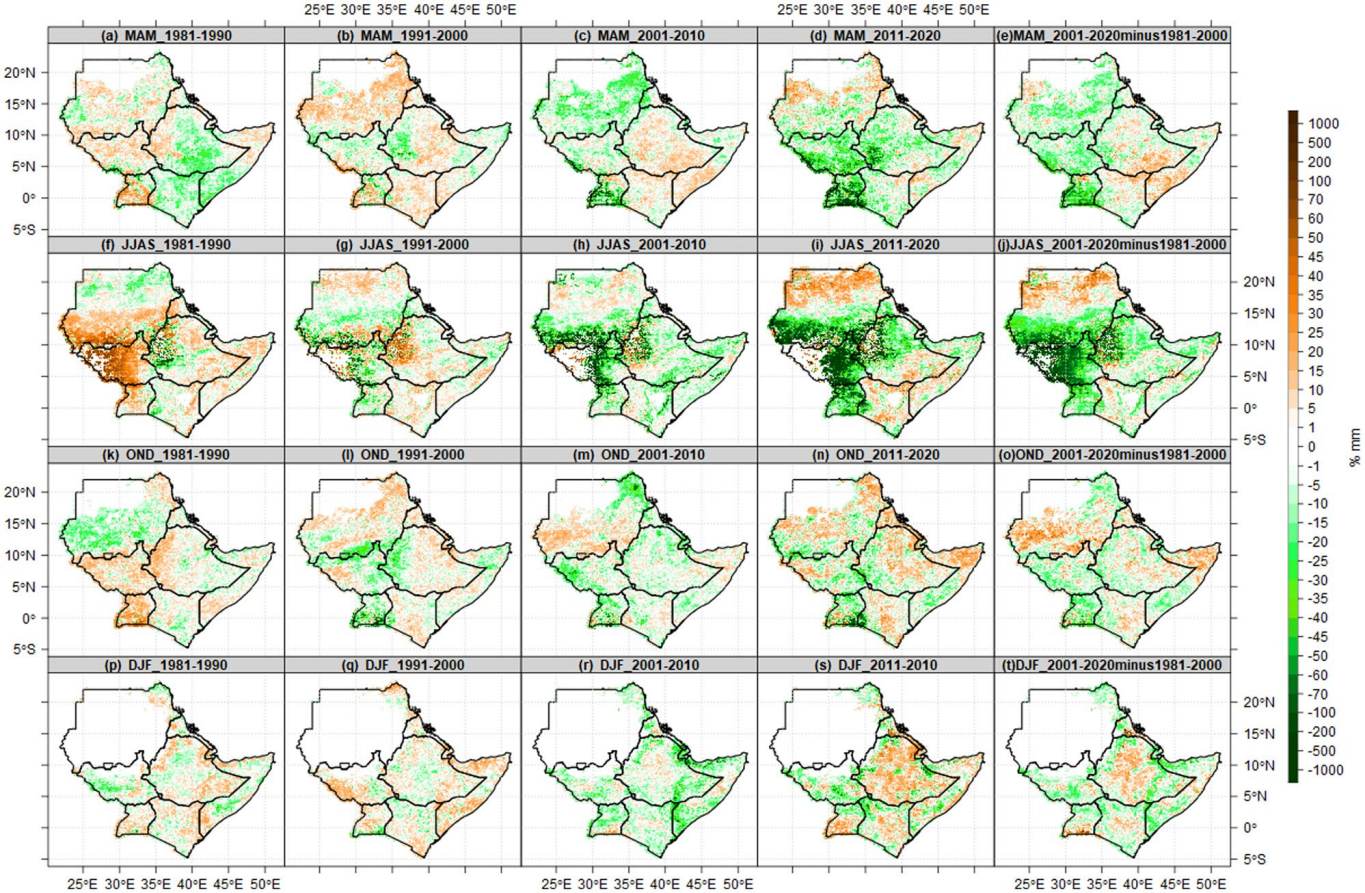
Changes in Seasonal mean state of Number of dry spells at 1 mm thresholds for the four decades (1981–1990, 1991–2000, 2001–2010 and 2011–2020), average of 40 years (1981–2020) and differences between current 20 year (2001–2020) and previous 20 years (1981–2000). The Brown color (positive values) indicates decreased dry spells, while green color (negative values) indicates increased dry spells.

Figure 10 illustrates the observed rate of change (%) in the seasonal mean state of the number of dry spells. As seen in the wet day’s analysis already covered in Fig. 10 above, the dry spell results once more reveal considerable decadal (10 years) variability of dry spells over the IGAD region. Most areas of the region had a 5–20% decrease in the number of dry spells during the most recent ten years (2011–2020). The MAM and JJAS seasons have observed a 40% decrease in dry spells in recent years (2011–2020). Sudan, South Sudan, and northern Uganda observed a decrease of 35 per cent in dry periods throughout the MAM, JJAS, and OND seasons. During the DJF, the region observed less than 15 percent decreased dry spells, particularly in Kenya and Uganda (Fig. 10s). In the 1980s, a 10–35% increase in wet spells was observed over most of Uganda, South Sudan, and western Ethiopia. During MAM, dry spells decreased by 5–10% in most parts of Kenya and Sudan, eastern Ethiopia, and southern Somalia. The 1980s observed a broad 15–35% decrease in dry spells throughout South Sudan, a rain belt in southern Sudan, and western Eritrea. Dry spells increased by 5–10% in South Sudan, western Ethiopia in the 1980s (Fig. 10a), southeastern Ethiopia, and throughout Uganda, Kenya, and Somalia in the 1990s (Fig. 10b). In addition, coastal and northeastern Kenya, southern and central Somalia, and most of Ethiopia experienced less than a 15% increase in the 2000s.

### Implication of wet days and dry spells on drought and floods patterns

**Figure 11:**
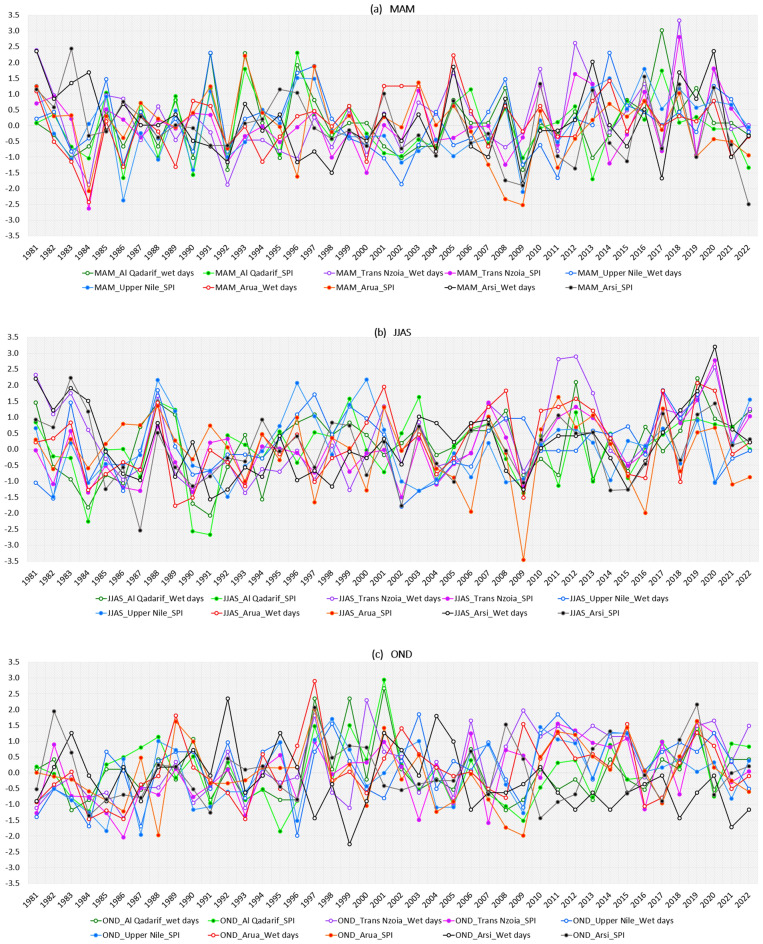
Inter-annual anomalies of wet days over 5 potential agricultural sub-regions (the wet days computed over entire pixels in El Gadaref state in Sudan, Upper Nile County in South Sudan, Arsi zone in Ethiopia, Trans Nzoia county in Kenya and Arua district in Uganda) reference to 1981–2010 bassline.

Figure 11 presents the Inter-annual variability of wet days’ anomaly over five potential agricultural and food baskets in the region. The wet days anomalies were compared with the SPI signal to assess the implication of wet days on drought/flood patterns over El Gadaref in Sudan, El renk in South Sudan, Arsi in Ethiopia, Trans Nzoia in Kenya and Arua in Uganda. In general, the statistics reveal that the majority of the IGAD region experienced exceptional(unusual) drought/floods coincided with exceptional(unusual) decreased/increased wet days anomalies. The yearly variation in wet days anomalies is high across five sub-locations. These results confirmed the devastation drought occurred in the 1980s and exceptional wet conditions in recent years (2011–2020). In other words, the patterns of increase/decrease in wet days and dry spell anomalies significantly contributed to extra-ordinary drought and floods events in the past. For instance, MAM 2018 recorded extra-ordinary(unusual) increased number of wet days over Trans Nzoia in Kenya. The OND season in 1997 was the wettest year in the last 41 over most parts of the region, especially over Arua in northern Uganda and the highlands of western Kenya. This is in line with the conclusions reached by Ayugi et al.^68^, who found that the wettest years were 1997 and 1998, whereas drought occurrences were observed in the years 1987, 2000, 2006, and 2009 for SPEI-3 and 2000 and 2006 for SPEI-12^68^. The JJAS 2018 over Arsi in central Ethiopia recorded the highest wet days anomalies since 1981. The wet days and SPI anomalies over Upper Nile in South Sudan and Alqadaref in Sudan behind 1988 widespread floods. The increase in wet days in recent decades (2011–2020) was driven by extra-ordinary increased wet days in 2018, 2019 and 2020, while decreased wet days anomalies in the 1980s were driven by dry conditions in 1983–1986 over most parts of the region. Similarly, the severity of drought events in 1983, 1984, 1985 over Sudan, South Sudan, Ethiopia, 2011 Somalia, and 2021 over most parts of the region accelerated by a decreased number of wet days and prolonged dry spells. The decreased wet days anomalies in 1990/1991 over Alqadaref in Sudan exacerbated the extreme drought conditions in the record. These findings are consistent with those of Zhang et al.^69^, who determined that the most severe drought occurred in the 1980s, particularly in 1984, when more than 4.5 million people in Sudan were seriously afflicted and needed aid. The decreased wet days anomalies from OND 2020 to OND 2022 triggerred five consecutive failed rainy seasons over most parts of the IGAD region.

### Projected changes in wet days and dry spells patterns

**Figure 12:**
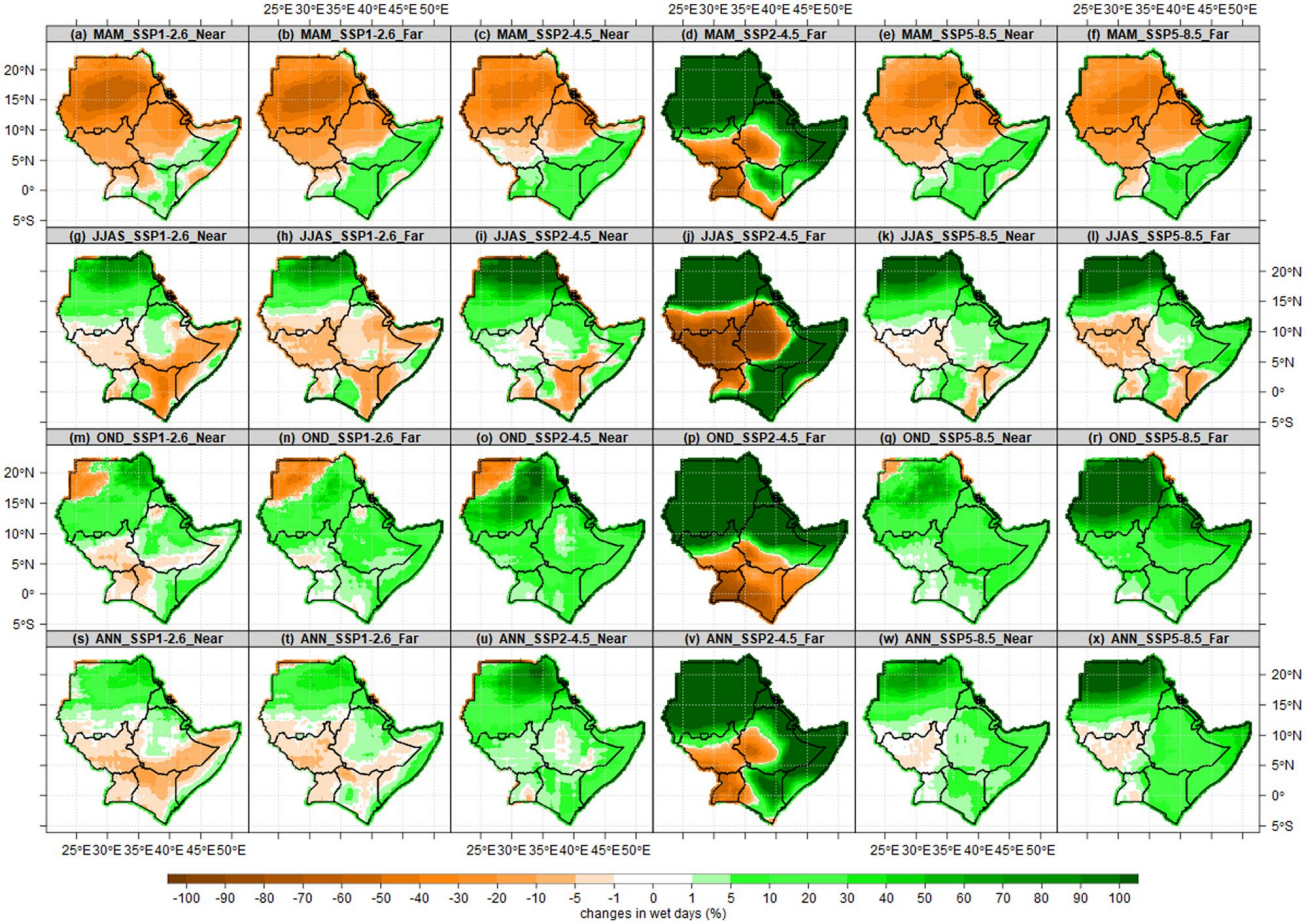
Spatial pattern of the projected number of wet days over the IGAD region is based on the CMIP6 ensemble mean for the near future (2021–2050) and far future (2071–2100) relative to the baseline period (1985–2014). The three future scenarios (SSP1-2.6, SSP2-4.5, SSP5-8.5) analyzed for MAM (first row), JJAS (second row), OND (third row) and Annual (ANN) in the fourth row.

Figure 12 presents the projected changes in wet days based on an ensemble of 10 of the best performed CMIP6 models over the IGAD region. The three future scenarios (SSP1-2.6, SSP2-4.5, SSP5-8.5) were analyzed for MAM (first row), JJAS (second row), OND (third row) and Annual (ANN) in the fourth row. The projected changes were carried out for the near future (2021–2050) and the far future or end century (2071–2100) relative to the 1985–2014 bassline(control) period. Except for the pattern of SSP2-4.5 for the far future), the future changes in wet days show a 10–20% decrease in wet days over Sudan, Eritrea, Djibouti and South Sudan under all three scenarios during the MAM season. Most parts of Kenya, Somalia, and southeastern Ethiopia, show an increased number of wet days. These findings varied from Ayugi et al.^70^ findings of a decrease in consecutive wet days (CWD) towards the end of the twenty-first century (2081–2100) relative to the baseline period (1995–2014) for MAM and OND over Kenya and Uganda. The discrepancy could be due to the models chosen, the baseline period, and the reference datasets. Similarly, our findings are consistent with Wainwright et al.^51^ findings of increased wet season rainfall. The JJAS season is projected to have a 30–40% increase in wet days over central and northern Sudan, a 10–20% decrease over South Sudan, the highlands of western Ethiopia and northern Uganda for both the near and far future. The 5–20% increase in wet days signal dominated the OND season over most parts of the IGAD region. On an annual time-scale, an increase in wet days is projected over Sudan, Eritrea, Djibouti, Somalia and coastal parts of Kenya, while a 20–40% decrease is projected over South Sudan, Uganda under SSP1-2.6 scenario and South Sudan under the SSP5-8.5. On an annual and across three seasons, Sudan is projected to have more than 50% increase in wet days under SSP2-4.5 scenarios.

**Figure 13:**
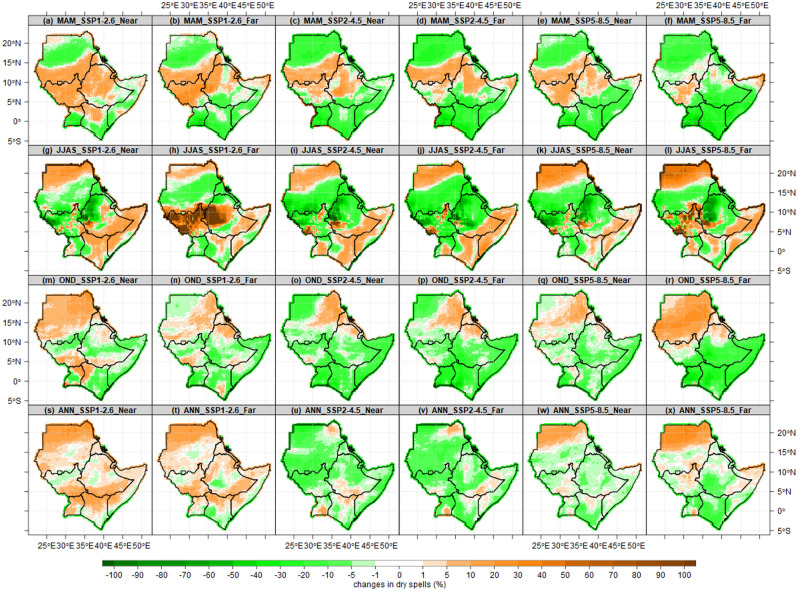
Spatial pattern of projected number of dry spells over the IGAD region based on CMIP6 ensemble mean for the near future (2021–2050) and far future (2071–2100) relative to the baseline period (1985–2014). The three future scenarios (SSP1-2.6, SSP2-4.5, SSP5-8.5) analyzed for MAM (first row), JJAS (second row), OND (third row) and Annual (ANN) the fourth row.

Spatial pattern of projected number of dry spells over the IGAD region presented in Fig. 13. Under all scenarios, projected changes in dry spells show a 10–20% decrease over central and northern Sudan, most parts of Uganda, Kenya, Somalia and Djibouti, while 10–20% increase over southern parts of Sudan, most parts of South Sudan, highlands of western Ethiopia during MAM season. The changes in dry spells over Sudan seem to suggest shrinking in the dry season which extends from October to June. In Extreme northern Sudan, ASALs in Kenya, and southern Somalia are projected to have 10–20% increase in dry spells as opposed to a study by Ayugi et al.^70^, while a decrease is projected over southern and central parts of Sudan and most parts of Ethiopia across all scenarios during JJAS. These patterns suggest the prolonged dry season of JJAS over ASALs in Kenya and southern Somalia. The eastern Sudan, consistently projected to have increased dry spells, decreased over most parts of Uganda, Kenya, Somalia, southeastern Ethiopia during OND. Also, JJAS and OND patterns suggest the expansion of the rainy season over the Red Sea coastal parts in Sudan and Eritrea. Annually, dry spells are projected to increase under SSP1-2.6, decrease under SSP2-4.5 and SSP5-8.5 over most parts of the IGAD region. It appears that a rise in the number of rainy days and a decrease in dry spells explains the enhanced rainfall signals found by Mbigi et al.^71^ over Uganda and Kenya, Ogega et al.^72^ over Lake Victoria basin as well as the growing trend reported by Alaminie et al.^73^ over Ethiopia's Upper Blue Nile Basin and Ngoma et al. over Uganda^74^.

## Discussion

In this paper, we have used criteria and thresholds in identifying wet days and dry spells characteristics. The possible number of wet days and dry spells is 91 days (12 spells) for MAM, OND and DJF, and 122 days (17 spells) for JJAS using CHIRPS and an ensemble of CMIP6 models across the IGAD region. The results show the majority of districts in Uganda, counties in South Sudan, zones in Ethiopia and highlands of western and Nyanza counties in Kenya observed the highest number of wet days. The mean wet days patterns proved the potential of these areas for sustainable food crops production and food security. The south-northwards and north-southward movement of ITCZ around the Equator suggests the highest (lowest) number of wet and dry days over the region. The mean state of dry spells is consistent with the earlier findings by Gitau et al*.*^40^, which explained the nature of variation in dry spells over Equatorial Eastern Africa. The highest number of wet days (lowest wet days) are recorded in areas where the MAM, JJAS, OND and DJF seasons are considered as the main rainfall season (dry climatology). The highest number of wet days explained the importance of JJAS for South Sudan, and Ethiopia, while MAM and OND for Kenya, MAM, JJAS, OND for the Uganda agriculture sector now and in the future. In addition, the results show, the majority of lands in the IGAD region are dry and susceptible to agriculture failure due to 2–4 consecutive dry spells, which are projected to increase in the future under SSP1-2.6, SSP2-4.5 and SPP5-5.8. The observed and projected prolonged number of dry spells over most parts of ASALs in the region explained why they experienced chronic food insecurity now and in future if no adequate measures/interventions will be in place. The mean wet days and dry spell patterns could explain the south-north timing of rainfall onset progression and retreat(cessation) from March to November Omay et al.^63^. No changes in the pattern of 1% probability of exceeding 2, 3 and 4 consecutive dry spells over northern parts of Sudan during the MAM season because these areas are dry climatologically and ICTZ never crossed the Sudan border this time of year. Most parts of Kenya and Ethiopia during OND and DJF recorded a 60–100% probability of exceeding 2, 3 and 4 consecutive dry spells because it is not possible for 7–28 days to pass without 1 mm rainfall thresholds. Since JJAS is the main season over South Sudan, northern Uganda, highlands of western Ethiopia, this explains why the probability of exceeding 2, 3 and 4 consecutive dry spells are less than 1%. This is supported by the study by Muluneh^75^ which found about 99 and 80% probability of dry spell lengths of 5 and 7 days in Babile and Haramaya districts of Eastern Hararghe, Ethiopia. Similarly, the patterns of 1% probability of exceeding 2, 3 and 4 consecutive dry spells covered all of Sudan during the DJF season. The increase in wet days over most parts of the region in the current 20 years (2001–2020) compared to previous 20 years (1981–2000) contributed largely to exceptional wet days in 2018, 2019 and 2020. This is in agreement with studies by Nicholson^76^, who reported a decline in rainfall over the East during the MAM season in recent decades. Increases in dry season rainfall over East Africa are also found by Wainwright^77^. The variability in East African wet and dry patterns historically has been linked to the influences of the atmospheric phenomena over the Indian Ocean^78,79^, the Pacific Ocean^43,44^ and the high variance of westerly winds over the central equatorial Indian Ocean especially during October–November^76,80^. At the regional level, the trend of wet days and dry spells of all pixels over the IGAD region observed to increase in wet days and decreased in dry spells across all MAM, JJAS, OND and DJF seasons. Similarly, at national levels, the trend of wet days and dry spells experienced a slight increase in wet days (decreased dry spells) during JJAS and OND. At the sub-national level, the trend of wet days and dry spells over El Gadaref state, Upper Nile state, Arsi zone, Tranz Nzoia county and Arua district confirmed the signals at countries levels with increased wet days and decreased dry spells especially during 2011–2020 decade. The patterns of wet days and dry spells in this study explain the decreased trend of total rainfall in the 1980s and 1990s which recovered in recent years (2011–2020), due to an increase in a number of wet days and decreased dry spells at regional, national and subnational levels. This pattern in line with the study by Wainwright et al*.*^77^, which reported that decline in the Eastern African Long Rains from the 1980s to the late 2000s. The Implication of Wet days and Dry Spells on Drought and Floods Patterns is proofed to be linked to an extra-ordinary increased number of wet days and decreased in dry spells. These results are in close agreement with the findings by Ayugi et al*.*^68^ that focused on the comparison of CMIP6 and CMIP5 models in simulating extreme wet and dry patterns over East Africa. For instance**,** the decreased number of wet days in 1983 and1984 seems to have played a role in the intensity of drought over South Sudan, Uganda. Also, the wet days patterns played a significant role in increased/decreased total rainfall and changes in rainfall trends.

Projected changes in a number of wet days and dry spells are not consistent across the IGAD region. The decreased number of wet days over Sudan and South Sudan during the MAM season under SSP1-2.6, SSP2-4.5 and SPP5-5.8 could be an indicator of the expansion of the dry season or desert belt southward by 2100. The likelihood of increased wet days patterns during OND under different scenarios may confirm the wet condition reported in CMIP5 over East Africa. The future changes in wet days and dry spells don’t occur coincided with increased/ decreased in wet days and increased dry spells length. These could be attributed to wet and dry biases signals in some models’ simulations and projections. The magnitude of changes in wet days over Sudan under SSP2-4.5 is a larger increase compared to other scenarios, which possibly could be related to the higher climate sensitivity in the medium scenarios (SSP2-4.5). Also, coincided increases/decreases in wet days and dry spells over most parts of the IGAD region, were found to be robust across the three scenarios. Again, the increases in wet days and decreases in dry spells ASALs in the region were present in all three scenarios, but were only significant (40–60%) over Sudan under SSP2-4.5. The observed and projected changes in a number of wet days and dry spells in the region play a significant role in rain-fed agriculture planning, necessary arrangements for supplementary irrigation, and early selection of the crop type and variety in the IGAD region.

## Conclusions

We conducted an analysis of changes in wet days and dry spells across eight IGAD member states over a 40-years (1981–2021), near future (2021–2050) and far future (2071–2100) under three socio-economic pathways (SSPs), SSP1-2.6, SSP2-4.5, SSP4.5 and SSP8.5. Changes in the length of wet days and dry spells periods in the IGAD region of eastern Africa have been observed with a shift to longer droughts (coincided with decreased wet days and increased dry spells length). The increased number of wet days and decrease in dry spell length in the recent decade (2011–2020) exhibited by extreme wet days in 2018, 2019 and 2020, while increased dry spells and decreased wet days in 1983 to 1986 exhibited 1983/1984 and 1985/1986 devastating drought over Sudan and Ethiopia. The shortened number of wet days and prolonged dry spells over ASALs in Kenya, Somalia and southeastern Ethiopia explained the reason for chronic food insecurity and water scarcity witnessed in recent years. MAM season, being a dry season, is projected to be drier over Sudan, South Sudan, and Ethiopia under both low (SSP1-2.6) and high (SSP5-5.8) scenarios. South Sudan is the most likely nation in the region to experience a 20–30% drop in the number of wet days during JJAS under SSP1-2.6, SSP2-4.5, and SPP5-5.8. For the OND season, the SSP5-8.5 scenario indicates an increase in wet days throughout the IGAD region of Eastern Africa by 2100. Findings from this study provide insight into risk of drought and floods associated with prolonged wet days and dry spells and changes in the mean state of wet and dry spells. In addition, it is offering a wide view for policymakers, rain-fed agriculture and food security interventions. Future work will concentrate on the implications of changes in wet days and dry spells on total rainfall, rainfall intensity and drought/flood patterns.

## Data Availability

The secondary datasets generated during analysis are available via request. The primary data are open access from Index of /products/CHIRPS-2.0/global_daily/netcdf/p05 (ucsb.edu).

## Code availability

We used open-source statistical tools ((Climate Data Operators (CDO) command lines, R-Package and Climate Data Tool (CDT)) and ArcMap10.4.
